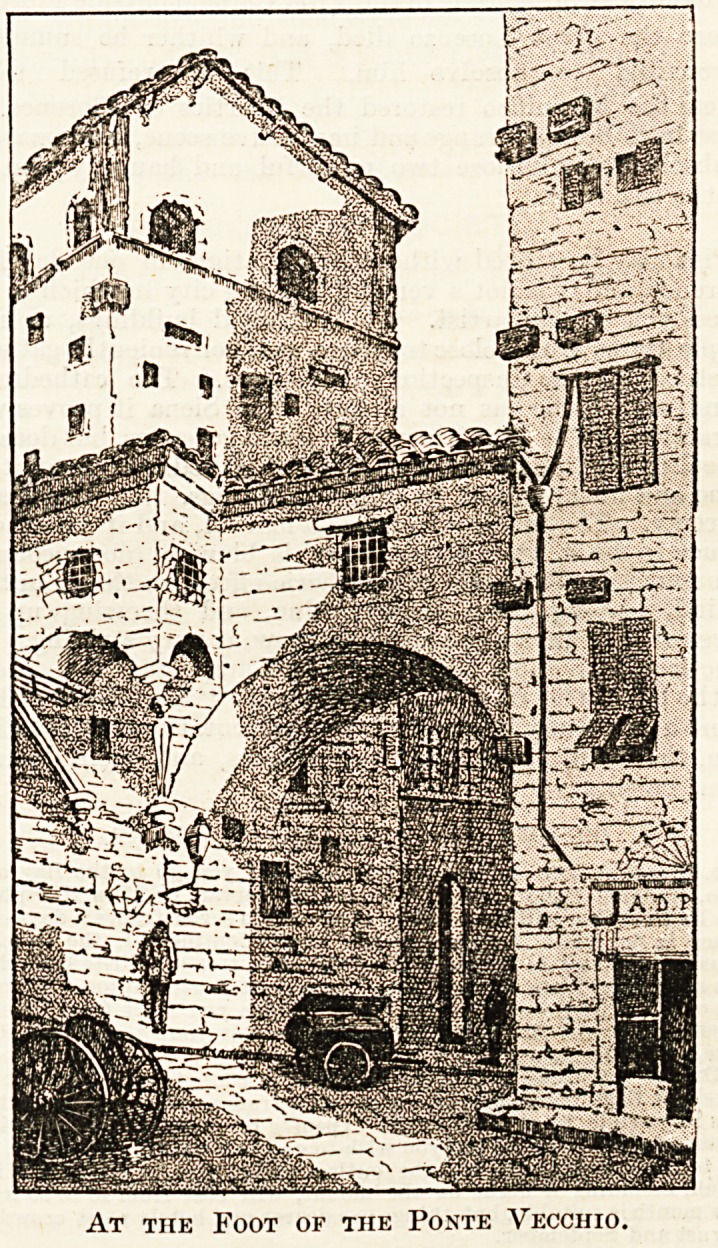# "The Hospital" Nursing Mirror

**Published:** 1899-06-03

**Authors:** 


"The Hospital" Nursing Mirror.

Being the Nursing Section of "The Hospital."

[Contributions for this Section of "The Hospital" should be addressed to the Editor, The Hospital, 28 & 29, Southampton Street, Strand, London, W.C., and should have the word "Nursing" plainly written in left-hand top corner of the envelope.]

### Notes on News from the Nursing World.

### APPROACHING MARRIAGE OF MISS HICKS.

We understand that Miss K. Phillippa Hicks, daughter of the Rev. Robert Hicks, late rector of Kirk Smeaton, Yorkshire, is shortly to be married to Mr Herbert Emmott Large, of Flintham Lodge, Oksey, Wilts, who formerly practised as a solicitor in Gray's Inn. Miss Hicks had the honour of being selected in 1885 by Miss Florence Nightingale, acting on behalf of the Princess of Wales, for service in the Soudan. During her brilliant professional career she has been matron of the Hospital for Sick Children in Great Ormond Street, and first lady superintendent of the Nurses' Co-operation, which she resigned on account of ill-health. Under her able management the co-operation achieved its present satisfactory position. We assure Miss Hicks that the numerous nurses who have worked under her at different times will not only be interested in the announcement of her approaching marriage, but will join with us in wishing her all possible happiness.

### ENTERTAINMENT TO LEEDS NURSES.

On Monday evening about one hundred nurses from the different hospitals and homes in Leeds availed themselves of the kind invitation of the Lord Mayor and Lady Mayoress (Colonel Harding and Mrs. Harding) to an "At Home" at their lovely house near Kirkstall Abbey. Waggonettes were sent to convey the nurses to and from their destination. During the evening selections of music were rendered at intervals, but one of the most enjoyable parts of the entertainment was a recital of some quaint reminiscences by Mr. Blakebow in the real Yorkshire dialect. The house was thrown open to the nurses, and an inspection of the different rooms that had at a remote time (about the year 1107) been inhabited by the monks connected with the abbey proved intensely interesting. Refreshments were served during the evening, and at ten p.m. the nurses returned to their various duties, feeling very grateful to their host and hostess for making the evening such a happy one.

### ANNUAL CONFERENCE OF THE AFFILIATED BENEFIT NURSING ASSOCIATIONS.

The sixth annual conference of these associations "for the Supply of Cottage Nurses on the Holt-Ockley System" was held, by permission of the Right Hon. A. J. Balfour, M.P., and Miss Balfour, at 10, Downing Street, on Tuesday afternoon, the Earl of Gainsborough, president, in the chair. Miss Broadwood gave an account of the work of the associations during the year past, and read a report from Miss Mocatta, matron of the Royal Hants County Hospital, commenting favourably upon the nurses she had inspected, and the character of their work. Subsequently Miss Broadwood stated her conviction that the most suitable nurse for cottage homes is a woman of the cottage class. The associations propose to start a special home for cottage nurses and to help forward this project Miss Broadwood appealed for financial assistance. The proceedings concluded with an address on the "Prevention of Typhoid Fever and Tuberculosis" by Dr. Christopher Childs.

### THE SALARIES OF NURSES IN THE TROPICS.

After a somewhat long interval an authoritative statement has been sent to us by the hon. secretary of the Colonial Nursing Association. In no case, we are glad to learn, is any nurse working under the auspices of the association offered a salary of less than £60 a year for private work, with board and lodging, and journey out and home provided; while all appointments made for hospital work are Government appointments, the salaries of which are fixed by Government, and vary, according to the size of hospital and the climate, from £100 to £250 a year, with the usual advantages accorded to all civil servants. In these circumstances the association should be able, and doubtless will be able, to command the services of ladies equal to the onerous duties.

### THE CHOICE OF A TRAINING SCHOOL.

The case of Miss Glaise, which is reported in another column, should serve as a warning to those about to enter the nursing profession to exercise the greatest caution in regard to the choice of their place of training. We do not propose here to deal with the extraordinary evidence givenin this case, but we would point out that no training in any such institution as the Cottage Hospital, Bedminster, could be of any service to those seeking responsible positions in the larger hospitals, or even wishing to become superintendent workhouse nurses. No better service was ever done to nurses than by the issue of the Local Government Board order defining the standard of training necessary for those who would become superintendent nurses in the poor-law service, namely, three years' training in a general hospital or infirmary, being a training school for nurses, and maintaining a resident physician or house surgeon. If that, which is now looked on as the standard, be compared with the training offered at the institution at Bedminster, the inadequacy of the latter for those who wish to become "trained nurses" will be apparent to everyone. In the case before the court the defendant won the day. The question put to the jury, however, had no reference to the sufficiency of the training. The verdict said that the defendant honestly believed that he was able to give "the training described in the letters, prospectuses, and advertisements," but it said nothing as to what that training was like. This brings us to the point again. When would-be nurses are selecting a training school they should take care that the training which they will get will render them "trained nurses" in the proper meaning of the word, and they must remember that if any dispute arises no court will take any notice of what they think or expect, but will go entirely by the letter of the bond.

### THE PROGRESS OF NURSING IN THE PRINCIPALITY.

There are several signs that nursing is making distinct progress in Wales. At Swansea there has just been opened an addition to the hospital, consisting of a nursing home of ample dimensions, providing accommodation for eighteen nurses. Each nurse will have a separate bedroom; there is a large dayroom on the ground floor; and bathrooms are on both floors. The building is connected by a corridor with the hospital. In quite another part of the Principality, at Cefn, near Llangollen, it has been determined by the parish council to employ a trained nurse. As the inhabitants are mainly colliers, brickmakers, and quarrymen, her services are sure to be often in request. At Trevor and Vroncyssylltau a nursing association is doing good work.

### WEST RIDING NURSES' ASSOCIATION.

A note in our last on "A Bankrupt Nurse" referred to the West Riding Nurses' Association as an unsuccessful venture. We are very glad to learn from the matron of the institution, Sister Bessie, that it is doing very well indeed. In fact, the matron states that through the past winter and spring there has been a greater demand for nurses than the association has been able to supply, and an endeavour is being made to increase the number. We are informed that Mrs. Fry worked at first as nurse on the same terms as the other nurses, and later on joined Sister Bessie as partner, but left 12 months ago.

### ANOTHER AMBULANCE FATALITY.

Further discredit has been thrown upon the manner in which the ambulance system is sometimes managed by an accident at Liverpool, resulting in the sacrifice of two lives. In this case the poor fellow who was being conveyed to the hospital in an ambulance and the driver of the vehicle were the victims, though it is fair to add that the death of the former was proved at the inquest to have been primarily due to the fractured skull from which he had been previously suffering. Still, it was probably accelerated by the accident to the ambulance, which was wrecked by a collision with a lamp-post, due to the horse bolting. It is stated that the unfortunate driver was competent, but it is certain that the horse was not, for he was blind in one eye. To use such an animal for ambulance work shows an extraordinary lack of consideration for the very persons who most need it. Moreover, although an officer appears to have been inside the vehicle, there is no mention of the trained nurse who should always accompany it.

### A NURSE AND HER CERTIFICATE.

Miss Christina Jackson had for two years been Queen's nurse at Blairgowrie, when she resigned and asked for her certificate. This is usually granted by Queen Victoria's Jubilee Institute in London upon the recommendation of the lady superintendent and the committee of the association under which the nurse has worked. After some delay, owing to the absence of one of the ladies of the committee, a motion recommending that Nurse Jackson should receive her certificate was assented to. When, however, Miss Jackson was given the document she found that the customary words "With satisfaction to the Superintendent and Committee" had been omitted, and she naturally wished to know the reason why. The committee refused to give her the explanation she sought, and she accordingly placed the matter in the hands of her solicitor. The case, which is exciting great local interest, will be gone into fully at a general meeting of the subscribers to the Blairgowrie Nursing Association, which will be held shortly for the purpose.

### SHORT ITEMS.

Thanks to the liberality of the members of the Stock Exchange, the council of the Royal Eye Hospital at Southwark are able to announce the opening of a ward which has hitherto been closed for want of funds. The opening ceremony will appropriately be performed by the chairman of the Stock Exchange on Monday afternoon.—The Duchess of Albany will, on Wednesday week, lay the foundation stone of a new wing of the Hospital of the Nursing Sisters of St. John the Divine, Lewisham. The wing will contain 30 free beds, and the total cost will be about £6,000.—In the current number of the *St. Thomas's Hospital Gazette* reference is made to the loss of Miss Christine Elinor Pollock in the "Stella." Miss Pollock left the hospital last June at the end of her four years' training. From St. Thomas's she went to the Women's Hospital, Euston Road. Her death, it is stated, "has been the cause of much sorrow," and "a particularly sad feature in connection with it was that she was shortly to be married."—On the Queen's birthday, immediately after the ceremonial parade at the saluting battery, Sir Francis Grenfell, Governor of Malta—after a short speech in which he referred very kindly to the work of the Army Nursing Sisters in Egypt—decorated Sister M. E. Harper with the Royal Red Cross conferred upon her by the Queen for nursing the sick and wounded during the recent campaign in the Soudan.—On Saturday a meeting will be held at Grosvenor House at half-past three p.m. by invitation of the Duke of Westminster, president of the National Health Society, when H.R.H. Princess Christian will present the diplomas, medals, and certificates of the society to successful candidates. The chair will be taken by the Duke of Westminster; and amongst the speakers who have promised to address the meeting are Sir Richard Thorne-Thorne, K.C.B. (chairman of the council), the Rev. C. J. Ridgeway (chairman of the Executive Committee), the Bishop of London, and Sir William Broadbent.—Mr. and Mrs. Walter Johnson have issued invitations for an "At Home" and garden party at The Cedars, High Road, Upper Clapton, on Thursday, June 15th, in aid of the North-Eastern Hospital for Children, Hackney Road. The programme of amusements includes pastoral plays, bands, games of all kinds, the ever-popular gramophone, and the new wonders of wireless telegraphy.—At the annual meeting of the Winifred Children's Convalescent Nursing Home on Monday, the Chairman, in moving the adoption of the report, laid stress upon the importance of skilful watching when instruments are used upon children, and stated that three of the children on their admission to the home were suffering from sores due to pressure in the wrong place.—The Duchess of Sutherland will open the annual sale of work on behalf of the inmates of the Royal Hospital for Incurables, West Hill, Putney Heath, on the afternoon of Tuesday, June 27th, at half-past two. The sale will remain open on the 28th and 29th. The charge for admission each day will be allowed in purchases.

### District-Visiting Nursing in Obstetric Practice.

By A. Worcester, M.D., Waltham, Mass.

(*Continued from page* 113.)

Henceforth, at least in Canada, district-visiting nursing will receive deserved attention; and it is greatly to be hoped that in the United States also more attention will be paid to this too neglected department of nursing.

There are, it is true, a few district nursing stations in some of our cities, and, doubtless, some of the visiting nurses in such employ have picked up some parts of the art. But there is no instruction given in this department of nursing in the large training schools. And so little is this great lack appreciated that graduates of such schools have offered their services as teachers of district-visiting nursing and as superintendents of the recently established stations in Canada. Such sublime self-confidence is ahead of the Irishman's who was sure he could play on the violin although he had never tried; but he did not offer himself as a teacher.

In the large training schools little change need be expected. They serve their hospitals admirably, and admirable hospital nursing is all that is there wanted. Hope for the professional improvement of nursing rests upon the development of the smaller schools, which, not being fast bound to the service of large hospitals, may follow more and more faithfully high and truly educational ideals. Fortunately, in the training schools connected with small hospitals hospital nursing cannot overshadow other branches of nursing. And as these small schools are rapidly increasing in number, and as their student nurses even now in this State outnumber the student nurses of the large hospital training schools, the time perhaps is not far off when graduate nurses will no longer rank themselves by the size of the hospitals which own their respective schools, but rather by the educational breadth and thoroughness of the training given therein.

In the more general training that is of necessity given in the smaller schools, neither obstetric nursing nor district-visiting nursing is slighted. Indeed, both branches are taught simultaneously; for a large part of the visiting nursing is for obstetric service, where is afforded the best possible opportunity for teaching to student nurses the art of obstetric nursing.

Having for the past thirteen years devoted much time to this kind of teaching I venture to submit this outline of what I try to teach.

The first principle of district-visiting nursing is the adaptation of service to the needs of the family, and thus securing the willingness of the family and patient to accept such service. This implies the carrying out of one's method of nursing only so far and so fast as can be done without provoking opposition.

### Preparation of the Lying-in Room and Bed.

There will always be found too much furniture in the room to be prepared for labour, as the room will have been used for other purposes. The nurse should have an idea of what the room should contain before she enters, and then, as she moves about, by making little changes here and there the room will get nearer and nearer to the best possible condition for labour. Getting out of the room the useless furniture I put first in the process of making ready, and storing it in that part of the house through which it will not be necessary to run a few minutes later. Rocking-chairs are a special abomination, but bric-a-brac is worse. A table with a bare top should be ready for the doctor's use when he comes. The top should be clean, or if altogether too dirty it can be made fit for use by spreading newspapers or clean cloth over it. The blinds should be open, if it is daytime, and newspaper curtains should cover the sashes if windows of opposite houses command the room. Plenty of light is more often lacking in the daytime than at night when artificial light can be had, and good light is sometimes of life-saving importance. The right side of the bed should be accessible and should receive the greatest supply of light. The protection of the carpet often fails. It is easy to spread papers over it but hard to make the papers stay in place. A low tub or some such vessel underneath the bed is the best protection. It can be pulled out to project a little where and when it is wanted.

A suitable narrow bed can seldom be had. Sometimes a little cot-bed may be found for the labour and the other bed used for the patient afterwards. The beds we find in district-visiting work are generally unsuitable in every way. The most expensive part of the bed is generally the mattress, not so much so among the people of some nations as of others. A feather bed is a very expensive thing; a hair mattress is also expensive, and the cheapest kind is too valuable to let it be damaged needlessly. It is very essential to protect the mattress from liquids soaking into it, so the best protective obtainable should be used for this purpose, especially on the right side of the bed. Stout wrapping paper is almost as good as a rubber sheet. Newspapers are better than nothing. The under sheet should be drawn smoothly and carried well under the right side of the bed. The draw-sheet should likewise be tucked well under the mattress on the right side and also fastened in some way on the left side of the bed. If not long enough to tuck under, it may be pinned to the mattress ticking. This much of the bed may be regarded as permanent. For its temporary protection a second draw-sheet should be put on so as to cover the bed and also the right side of the bedstead. In each draw-sheet should be folded either rubber sheeting or some sort of waterproofing.

### Preparation of the Patient.

Braiding the hair is quite necessary, especially if the hair is plentiful. When it comes to cleaning the patient it must be remembered that more than half find themselves unable to take care of themselves as pregnancy advances, and to have a young nurse come in and urge a bath upon the patient as preparation for the doctor's visit may offer great violence to her feelings. If tact is used the patient will probably be grateful to be put in order for the doctor's visit. There is no necessity for antiseptic treatment of much of the body. It is impossible to conduct an obstetric case in an absolutely aseptic manner. The body can be made aseptic only by violent chemical agents, such as permanganate of potassium and oxalic acid. Soaking in these agents, after soap and nail-brush work, will produce an aseptic condition. But when it comes to sterilisation of the vagina and vulva by such extreme precautions as are taken in operating-rooms on patients under ether, in obstetric cases such treatment cannot be thought of. It is sufficient to clean with soap and water between the hips and knees, especially the vulva, inner thighs, and nates. Then cloths soaked in corrosive sublimate, 1 to 1,000, should be pressed against these parts instead of scrubbing them. The cleansing enema must not be forgotten. If washed out beforehand, the rectum will at the last discharge only mucus.

The nightdress should be well rolled up and pinned under the right arm. If a vest is worn it should also be fastened up in the same way. There is little comfort in a clean nightdress after labour if the vest is soaked. The dressing of the patient is completed by tying, at the right-hand side of the patient, a sheet folded over the waistband.

(*To be continued.*)

### The Nursing of Phthisis in Sanatoria under the "Open-Air" System.

By the MATRON of the National Sanatorium for Consumption, Bournemouth.

### II.—THE QUESTION OF DIET.

In a modern sanatorium for open-air treatment of consumption the chief duties of the nursing staff may be summed up under the heads of management of the windows indoors and of shelters in the grounds; attention to the patient's cleanliness, diet, weight, and temperature, to the instructions of the physicians as to the regulation of the amount of time spent by each patient in the open air, and the physical exercises prescribed for each individual; and the proper education of patients as to their personal hygiene and the disposal of all expectoration. The nurse must also be prepared for the special emergencies of phthisis, such as sudden attacks of hæmoptysis, asthma, and heart failure, and, in the later stages of the disease, for signs of brain mischief.

Taking first into consideration the question of diet, the so-called "over-feeding" spoken of as practised in the German Sanatoria is not advocated by the best authorities in this country. They consider that the patient's digestive apparatus is in many cases unable to cope with the sudden call upon it, and that the over-taxed system is very liable to become so disorganised that the treatment only results in a general breakdown and inability to assimilate ordinary food, thus adding the aggravation of acute dyspepsia to the other general symptoms. In this sanatorium we find that patients do best if they are not allowed to take extras in the shape of solid food between meals. Living as they do out of doors, their appetites become keen in the intervals between meals, and they attack beef and mutton with a hunger to which they have in very many cases long been strangers. Variety of well-cooked food is an essential, and it has even been said that the diet in a modern sanatorium should range from the "fever diet of a hospital to the elaborate dietary of a first-class hotel."* The dainty dishing up and serving of all meals is a very important factor in helping the flagging appetite, whilst the knowledge, carefully instilled by the nurses, that increase of weight is looked upon by the doctors as one of the chief signs of improvement in their condition, and that that increase can only be brought about by the consumption of food, helps many at first to conquer disinclination and really eat a good meal, until by degrees they find appetite returning and thoroughly enjoy their food. The following dietary table suitable for a charitable institution such as this may be useful to nurses, serving as a guide to the limits between which variety without extravagance may be found. For breakfast, patients should always have the choice between tea, cocoa, and hot milk. Boiled eggs, porridge with milk, cold meat, and bread and butter should be always on the table, with the addition on different mornings of hot sausages; ham; bloaters or fish of some description; fried bacon or stewed kidneys.

For dinner we may have roast topside of beef, roast mutton, stews of fresh beef and mutton, boiled beef and mutton, and beefsteak pies and puddings, there always being a choice of at least two different dishes, besides fish for those unable to take butcher's meat. As puddings, there should be two days a week the milk variety—rice, sago, and tapioca, and on the other days suet puddings with either currants, rhubarb, fruit, or treacle, jam rolls or tarts, plum puddings and baked currant puddings; there must always be in addition a milk pudding on the table.

For tea the patients may have tea, cocoa, or hot milk, brown and white bread and butter, stewed fruit or eggs, ham, cold bacon, or fresh meat, jam or marmalade, with the addition of cake occasionally. Supper will consist of hot and cold milk, cocoa, porridge, or soup, and bread and butter. Special diets of poultry, &c., will be given when ordered by the medical men.

Vegetables, of course, vary according to the time of year. Stout, ale, and all stimulants must be always reckoned as an "extra," and only given when medically ordered. All those who can take it without discomfort should have milk with their midday meal. The patients here are encouraged to eat as much butter and fatty food as possible. The hours for meals in this sanatorium are: Breakfast, 8.30 a.m.; dinner, 12.30 p.m.; tea, 5 p.m.; supper, 8.30 p.m. We find that a tumbler of hot milk at 10.30 a.m. and 3 p.m. does not interfere with the appetite for meals, and is generally much appreciated. Extra milk is also given during the night; each patient drinks at least two pints of milk a day, besides consuming a large quantity in puddings, with porridge, &c. (We boil or sterilise all milk before using.)

Dyspepsia is a complaint from which many of the phthisical suffer, especially those of the servant girl class, and here let me remind the nurse that the cause is very frequently to be found in unsound teeth. It is hopeless trying to "feed up" a patient whose teeth will not allow her to masticate her food thoroughly; she will probably bolt it in lumps to escape a scolding for not eating it, and thus pave the way for chronic dyspepsia, provoking the very malnutrition and emaciation which it is our aim and object to avoid. In a sanatorium this will naturally come under the notice of the resident medical officer, but private nurses must always draw the attention of the doctor in charge of the case to complaints of indigestion and neuralgia, both of which generally yield to suitable treatment if attacked before they are allowed to become chronic.

In private work with consumptive patients the nurse will find that much tact is required in dealing with this question of diet. Patients are so liable to fancy that they cannot take ordinary food, and instead of persevering in their attempts, fritter away their digestive powers on slops and patent foods until they lose all power of assimilating ordinary diet. In sanatoria where there are many patients there is usually a wholesome spirit of rivalry and sympathy at meal times, one encouraging another by example and precept to take reasonable quantities of food with a view to an "increase" at the next weighing. The weighing is a matter of much importance, for it is of great prognostic service to the physicians. The patients should be regularly weighed once a week in the evening, in their night clothes, just before going to bed. Weighing in ordinary clothing is most unreliable, as even if care be taken that the same top garments are worn the underclothing must of necessity vary, and it is just that variation of a pound or two from week to week which is so important from the doctor's point of view. The nurse will find that her best plan is to have the weighing machine in one of the night wards. The patient should come to it dressed only in night-clothes, dressing-gown, and slippers, both of which latter articles can be slipped off as she steps on to the weighing machine. The weight recorded on the dial should be written down by the nurse in the book provided for the purpose immediately and on no account committed only to memory—memories are proverbially untrustworthy—and it cannot be impressed upon the nurse too much that it is the exact weight with its exact fluctuations which the medical man requires for his knowledge of the case. A steady rise of a few pounds week by week is a most hopeful symptom. It generally proves a better sign than a fluctuating weight, even if a larger amount be gained in some odd weeks.

* "Sanatoria for Consumptives," Walters, 1899.

### Exhibition of Nursing Requisites in Berlin.

By Our Special Correspondent.

One of the largest and most interesting exhibitions of nursing requisites that has ever been held on the Continent is now daily attracting large crowds of visitors to the famous Philharmonic, in Berlin. The purpose of the show, which was opened in the presence of the Princess Elizabeth of Hohenlohe, and will not be closed until the 18th inst., is to afford the general public an opportunity of making themselves acquainted with the principal methods of aid employed by the nursing profession at the present time. Some idea of the importance of the affair may be gathered from the fact that it was organised, under the special patronage of the Emperor and Empress, by a committee including Professors Virchow, Leyden, Bergmann, Gerhardt, Gusserow, Heubner, Olshausen, Senator, and Waldeyer, Drs. Leuthold, Lazarus, Coler, Ewald, and Heyse, and many more of the most distinguished German surgeons and physicians of the age.

Among the most striking features of the display is a fine representation of a thoroughly up-to-date hospital ward of 16 beds. This splendid model occupies a space of over a thousand square feet, and in connection with it is a large operating-room, which is likewise fitted up with all the most modern improvements. The nurses and patients are represented by life-size wax figures, and each of the former is dressed in the uniform of a different German hospital.

In the same part of the exhibition there is also on view one of Döcker's transportable barrack wards for consumptives, such as have been erected in considerable numbers on the Grabow Lake, near Oranienburg, and in Jerusalem; and several of the wall spaces are fitted up as private sick-rooms, with the object of showing how such apartments should be appointed and arranged in certain cases of illness. The bed in one of these rooms is occupied by the wax figure of a mother with her newly-born child in a cot by her side; and in another chamber is a representation of a little girl under treatment for diphtheria.

An exhibit equally worthy of note is that made by the Deutscher Frauen-Verein für Krankenpflege in den Kolonien. Their extensive stand is so arranged as to represent "a sick-room in the Tropics," and in front is an excellent model of the hospital at Togo. Another well-known nursing union—the Vaterländische Frauenverein—occupies three commodious tents on the stage, where it displays a large variety of utensils and conserves employed by the profession on the field of battle; while the Verein für jüdische Krankenpflegerinnen has fitted up its stall after the manner of a nurse's room in one of the leading Jewish hospitals of Berlin; and the Frauenverein für Irrenpflege in den Colonien gives a good representation of a ward in a Colonial *maison de santé*.

Quite a feature of the show is the display made by the school of cookery carried on in connection with the celebrated Lette Verein. This institution, which is under the patronage of the Empress Frederick, has on view many examples of its cooking for the sick, and several of the students daily add to their number in the presence of visitors to the "Ausstellung."

The organising committee hope, as a result of the exhibition, to be able to establish in Berlin a permanent museum.

### Wants and Workers.

"H. S.," 5, Winchester Villas, High Street, Walton-on-Thames, would be glad to hear of a comfortable cottage home near London where an aged woman, requiring kindly constant supervision, could be received for 15s. a week.

### The Maternity Charity and District Nurses' Home, Plaistow.

On Monday the annual meeting of the Maternity Charity and District Nurses' Home, Plaistow, was held, by the kindness of the Hon. W. Lowther, at Lowther Lodge, Lord Robert Cecil in the chair. The Bishop of Colchester and the Archdeacon of Essex were both present, and would have spoken on behalf of the charity but there was some little delay in opening the proceedings, and they had to leave early. After a few words of introduction from the Chairman, Mr. Skewes Cox, M.P., moved the first resolution: "That this meeting welcomes the fresh development which has been assured by the magnificent gift of the new building, and that those present, while offering their hearty thanks to the anonymous donor, pledge themselves to promote its success in every way they can, especially by providing suitable furniture." Dr. Angus Kennedy, one of the hon. staff of the charity, seconded, and it was carried unanimously. Mrs. Charles Egerton proposed the second resolution, "That it is essential in the interests both of the patients and the nurses to increase the permanent nursing staff, and that this can most effectually be done by securing a larger number of annual subscribers." Alderman Phillips seconded the resolution in a very forcible speech, citing instances from personal experience of the great value of trained nurses, especially maternity nurses, in the homes of the poor. The report of the charity states that in order to meet the allegation of local medical men that the midwifery practice was injurious to their interest the small fee usually charged for attendance has been discontinued. All patients nursed now receive gratuitous service, but none possessing an income of more than 21s. a-week (unless circumstances warrant the exception) are eligible as patients. Doctors are supplied at their request with monthly nurses free of charge, either for the confinement or for daily visits, provided there is a nurse at liberty.

### Appointments.

Jessop Hospital for Women, Sheffield.—On May 25th Miss Minnie P. Walton was appointed Charge Nurse. She was trained at the York County Hospital, and the British Hospital, Endell Street, for maternity training. She also possesses the L.O.S. diploma. Her previous appointments were sister of the children's ward, York County Hospital for eighteen months; sister of a male medical ward at Dundee Royal Infirmary for three and a half years; and night superintendent of the Halifax Royal Infirmary for three months.

Borough of Hastings Sanatorium.—On May 10th Miss H. A. Matthews was appointed Matron. She was trained at St. Marylebone Infirmary, Notting Hill, London, W., and has been charge-nurse for three and a half years at Eastbourne Sanatorium; sister at Allt-yr-yn Hospital, Newport, Mon.; and matron of Isolation Hospital, Belper, Derbyshire.

### Death in Our Ranks.

We regret to announce the death of Emily Hutchinson, sister at the Strangers' Hospital, Rio de Janeiro, from yellow fever, May 3rd, 1899. She was trained at the Manchester Royal Infirmary. After gaining her certificate she went as sister to the Lewisham Infirmary, and resigned that post to take up work in the above hospital, where she had been for the last seven months.

### The Nurses' House at the Hospital for Sick Children, Great Ormond Street, W.C.

A CHAT WITH THE SECRETARY.

THE extension of The Hospital for Sick Children is now practically completed, and I had the opportunity last week of inspecting the new part of the building in Great Ormond Street under the auspices of the matron and the secretary. In the course of my visit I asked Mr. Adrian Hope to tell me the story of the valuable addition which has been made to the hospital.

"If you wish me to begin at the beginning," he said, "I found, shortly after my appointment as secretary, fourteen years ago, that the committee of the hospital were face to face with a very serious difficulty, namely, the want of proper accommodation for the nurses."

"Had the accommodation always been bad?"

"Yes, the site provided no means of supplying what is so necessary for the nurses—I mean a bedroom each. On the other hand, just at that time the owners of the adjoining property, the Roman Catholic Hospital of St. John and St. Elizabeth, began to agitate the question of rebuilding upon their site and extending the hospital into their garden."

"If they had carried out their scheme you, I gather, would have been absolutely unable to extend the Hospital for Sick Children?"

"Undoubtedly. It was the knowledge of this fact that made us anxious to come to an understanding. For some years, however, the negotiations could not be concluded owing to the many difficulties in the way, but at length, in 1897, an agreement between the owners of the Hospital of St. John and St. Elizabeth and the trustees of this hospital was signed. In 1898 we purchased the adjoining property for the sum of £30,000."

"What were the inducements for the purchase?"

"The great inducement was that the Roman Catholic convent was a building containing forty cubicles, which would at once go far to practically solve the very difficult problem of providing decent accommodation for our nurses by giving us forty separate bedrooms for them. In addition to the forty bedrooms we found that we should also obtain possession of a nurses' sitting-room, a dining-room, a reception room, in which the nurses could see their friends, a sitting-room for the matron, and other valuable accommodation."

"All of them most essential."

"But besides all these rooms, the purchase included a garden of about half an acre, in which our little patients can enjoy, at the time they so urgently require it, air and sunshine. Until the extension was secured the Hospital for Sick Children--the first children's hospital established in the British Empire, and the mother of children's hospitals—was in imminent peril of losing its light and air by the new buildings which, failing the purchase, would certainly have been erected by the Roman Catholics. In that event, too, the utility of the out-patient operation room and dispensary would have been destroyed."

THE HOSPITAL GARDEN.

"No doubt," continued the Secretary, "£30,000 seems a very large sum to expend when the only apparent result is to provide accommodation for the 40 nurses; but the removal of the nursing staff to the new quarters has made it possible to open a whooping-cough ward with 16 beds."

"I suppose that this was very much needed?"

"Urgently. We do not propose to take in a great number of ordinary cases of whooping-cough. That, as Dr. Barlow, our chief physician, pointed out a couple of years ago, when the question was raised, would be a very unwise procedure. But there are many cases of whooping-cough with complications of lung disease and of pneumonia which we have not unt now been in a position to take care of."

"So that the sum of £30,000 is not really to be spent on the nurses' house?"

"I think it seems but fair to divide it, and to say that the nurses' house should only be credited with absorbing half, the other half being counted against the new whooping-cough ward, the garden for the children, and the preservation of the light and air, so indispensable for a children's hospital."

"I presume that the £30,000 does not cover the adaptation of the convent to the requirements of the nurses?"

"No; the committee have spent an extra £3,000 or £4,000 in this work, which embraces a new system of drainage, a bathroom for every seven nurses, the introduction of the electric light, a proper system of ventilation and heating, and suitably furnishing the rooms."

"The amount you name seems very modest for the purpose."

"A great factor of economy has been that most of the work of adapting the new quarters to the needs of the nursing staff has been carried on under the superintendence of our consulting engineer, Mr. Charles Walrond, by our own works department, employing labour direct, and obtaining the different materials on strictly trade terms. This has ensured the best of workmanship and a saving of the profits that would have to be earned if the work had been done under contract."

"Apart from this reason, you would justify the extension in the interests of the nurses?"

"The claims of the nursing staff for proper accommodation—*i.e.*, at least one bedroom, a proper dining hall, and a suitable sitting-room where the nurses may rest—are so paramount that no hospital committee can be considered to have discharged their duty unless every effort has been made for its provision."

Mr. Hope gave an instance of the want of a reception room a little time ago, when the relative of a nurse called to acquaint her of the death of her mother. In the absence of a reception room, he had to break the sad news to her in the public hall of the Hospital.

The Nurses' Sitting-room.

At the meeting of the governors at which the extension was sanctioned, Mr. Edmund Owen, the senior surgeon, mentioned an episode which is worth repeating here. About half-past nine on Wednesday morning he was going from the entrance of the hospital to the theatre to perform a serious operation on a child, in company with one of the officials, and as they passed along a passage he heard music and singing in one of the rooms.

Mr. Owen said to the official, "What is the meaning of this unseemly merriment? Mothers are coming by with their children, and it sounds heartless if music and singing are heard." The reply of the official was, "The nurses have dined, and are just going to bed. It seems hard that they cannot enjoy a little music before they do so. Even night nurses require some relaxation." Mr. Owen added that he had never again heard any sounds of music at half-past nine in the morning, and he felt that he was hard-hearted and cold-blooded to have raised any objection. In the new house, nurses may enjoy music and singing without the fear of misconstruction.

Continuing his remarks on this subject, the Secretary said: "The women who labour devotedly and risk their lives in hospital work, deserve the best treatment which it is possible to give them. No false economy in the way of not providing them with every reasonable requirement should be entertained. Nurses do not want luxury, nor can committees afford to offer it, but comfort and privacy are essential to their work. If the nurses have no sitting-room where they can throw off the claims of their arduous profession, no privacy in their bedroom—and until now they have been sleeping six or more in rooms simply divided off from one another by curtains—their health will suffer, their *morale* will be lowered, and the work of the hospital must be inefficiently performed."

"Do you think that this view of the position has always been sufficiently grasped?"

"In past years I am afraid that hospital authorities have been content with endeavouring to make their wards as perfect as possible, and have left the nursing staff to get along as best they could. Fortunately, there has been a great reaction on this subject, and the tendency to-day is to recognise the responsibility attaching to the provision of proper accommodation for the nurses, and not to shrink an expenditure of money which the generous public has been induced to give for the nurses as well as for the patients."

"And how far has the public supported you in your appeal for the £30,000?"

"It has contributed £10,000."

"So that you still want £20,000. Not a vast sum."

"Only 40,000 half-sovereigns. We ask people who do not miss the price of a stall at a theatre, or that of a pair of twelve-button gloves, to come to our aid and minister for once as generously to sick children as they do often to their own pleasure."

There would, I think, be little hesitation among the charitable section of the community to assist the Hospital for Sick Children in the present circumstances if they saw how the outlay on the extension has been distributed. In no detail has luxury been attempted; at present, for lack of wherewithal, the corridors of most of the floors are uncovered, and the dining hall is still without chairs and tables. But both the sitting and bed rooms are tastefully and comfortably furnished. The general sitting-room looks pleasantly on to the charming garden. All the arrangements as to baths, heating, and the supply of water, are excellent. The bright, clean appearance of everything indicates the care which has been bestowed upon the work by all concerned. And lest any member of the nursing staff should be tempted to waste the electric light, it has been placed under the control of the nurse sister, who switches it on and off at a given time.

### Echoes from the Outside World.

### AN OPEN LETTER TO A HOSPITAL NURSE.

I AM afraid that a good many English people, women more especially, have not the slightest idea of the meaning of good manners. On Sunday some of the members of the Royal Family went to the Zoological Gardens in the afternoon. The visit was intended simply for pleasure, and no State of any sort was observed. For some time the Prince of Wales, accompanied by the Duchess of York, dressed in electric blue, succeeded in walking about amongst the people unrecognised except by a few friends, who naturally acknowledged their presence and then passed on. But at last, probably betrayed by a curtsey on the part of a lady saying good-bye to the Prince, the whisper as to the identity of the pair went from one person to another, and in a few minutes a crowd of gaping, staring, well-dressed women, with a few men here and there, had almost hemmed in the Prince and his daughter-in-law, and so persistently followed them that they had shortly to leave. If costers from the East-end had behaved in this way one would have felt vexed, though vexation might have been tempered by the reflection that they knew no better. But that people in a good position should act like snobs makes one feel horribly ashamed of one's countrywomen. No wonder the Royal Family in England so seldom walk about amongst the people, for there is every reason why they should not. I remember a few years ago when the Prince and Princess of Wales went to stay with the Duke and Duchess of Devonshire at Eastbourne, they were so mobbed that they had to quit the town. Apparently, matters have in no way improved since then.

HAVE you ever seen the folks going to the Derby? I do not mean the class who go respectably and decorously by train, but the East-end and publican element who drive down "in style." This year I happened to be staying with some friends who live on the high road to Epsom, and they made quite a feature of the event, had bunting put along the wall, seats arranged behind, and asked a party of friends to come and "see the fun," and stop to a cold collation. Such wonderful garments the women wore! Velvet blouses, trimmed with sequin trimming looked a trifle hot on such a broiling day, but were evidently thought quite correct. A skirt of one colour, a bodice of another, and a hat of a third, was apparently "distingué" when worn by a fat woman with a red face, seated in a very small pony "shay." A conspicuous turn-out was a hired brougham, wherein, facing the two horses, sat three sisters, all getting on for forty-five, and all weighing about twelve stone. Fitted tightly into the same seat, they were attired alike in cerise silk dresses trimmed with white, white Gainsborough hats with pink bows and heaps of white feathers, and white gloves. The two very young men were in white flannels, with pink ties, and pink ribbons round their straw hats. And every face looked pink with satisfaction at the beauty of the "tout ensemble"!

THE statement that the Countess of Warwick "serves behind the counter," in the daily papers, is not, it seems, quite accurate. There is no counter in the shop in New Bond Street, and Lady Warwick simply uses the establishment as a convenient place in which to receive orders for work. One of her objects in announcing that for the next month or two she would be present on certain days was that those who are interested in the question of women's employment in general, as well as in needlework, might know where they could find her if they had any problem they wished to chat over, or any development of a present branch of feminine labour which they could suggest. For Lady Warwick is an uncommonly busy woman, and also possesses an unusually good business head. At present she has a hostel at Reading where a number of girls are taught agricultural pursuits; she is going to bring out a monthly paper dealing with agricultural questions; and, as soon as she can, a girls' model lodging-house, with a club attached, is to be opened to accommodate the girls who belong to her School of Needlework. It is no longer practicable for the girls to live in the little cottages of their Essex villages, and consequently about thirty of them have been brought up to London so that it may be possible for the founder of the School to personally supervise their work when necessary. Their number will be doubled directly the model lodging-house scheme has been realised. When one remembers that all these ventures are superintended and planned by a woman who also takes her place in London society and plays her part in the functions of the hour, it makes one realise that it is not only the poor who work hard.

TALKING of society, one of the comparative novelties of the season is to be a married women's ball. Amongst a certain "set" the invitations will be looked for most eagerly, because the women who, although they have become wives and mothers, see no reason why they should not be as young and as skittish as ever, like the idea of having the field all to themselves, with no young *débutantes* to attract the older men and leave only the youths to them. The men, I am told, always like to be asked to the married women's ball, because they say that "it's jolly to be able to flirt as much as ever you like with no possibility of any watchful mother or eagle-eyed old chaperone inquiring—in the politest of language, of course—whether you have any serious intentions." The husbands, who may object to this proceeding, evidently do not count.

RATHER an amusing advertisement has been appearing in some of the London papers for several days. A lady wants a nurse (not hospital-trained) to take charge of a little girl of three. She insists that she must have no fringe, which is not altogether an unusual stipulation, though a really neat style of head-dress on the part of an employé daily becomes more desirable, as it becomes more difficult to obtain. But the essence of the advertisement lies in the words, "Required a respectable and *respectful* young woman." Poor lady! How visions rise before one of the pert and forward damsels who have had their excuses all too pat, the "bits of their minds" far too ready for distribution, whilst "Ma'am" and "Sir," "Mistress" and "Master" hid themselves away, and the air of "Hail fellow, well met, good-as-you-any-day" was all-pervading. Alas! I fear the times have changed, my dear lady, and the old-fashioned respectful servant has gone with them. A different class has arisen, with the spread of education, and though more difficult to manage because employers go on the old lines, instead of learning to run along the new ones, the new helpers have their good points, which we shall probably learn to appreciate more when we know them better.

VENTILATED hats, ventilated macintoshes, and ventilated gloves are all well known to us. But it has been reserved for a Montreal man to invent a ventilated boot. The air is pumped into the heel through a little valve, and so distributed that it plays all along the sole and also up the stocking. The inventor claims that the feet in this manner are kept dry and cool, and he may be right, but I doubt whether the notion is likely to be looked upon with favour by most women. The sole must naturally be a thick one to allow of the air grooves being inserted, and consequently the boots will appear clumsy, an unpardonable fault in the eyes of the woman who has a pretty foot—and also of the woman who wants to make out that she has.

### Legal Intelligence.

### A QUESTION OF TRAINING.

A CASE was decided at the Bristol County Court on May 18th which is of considerable interest in regard to the relations which exist between probationers and those on whom they depend for their training. The action was brought by Miss Lydia Glaise against Dr. W. L. Christie to recover £25 damages for alleged fraudulent misrepresentations and breach of agreement. According to the account given in the *Bristol Mercury*, Mr. Inskip, in opening the case, said the plaintiff, Miss Lydia Glaise, was now training as nurse at the Bristol General Hospital, and the defendant was Dr. William Ledingham Christie. The action was brought to recover damages—first, on the ground that defendant had dishonestly and fraudulently induced the plaintiff to pay ten guineas under the belief that she would receive proper training at a cottage hospital, supposed to be conducted by him at Bedminster, and the other ground of action was that the defendant undertook to provide her with efficient training and failed to do so, thus committing a breach of contract. Plaintiff was filling a situation in Yorkshire, and in the month of January her attention was attracted by an advertisement in THE HOSPITAL asking for probationers and nurses for training, inquiries to be made of "The Matron, Cottage Hospital, Bedminster." Plaintiff applied to the matron, and received in reply a letter and a prospectus. There was no doubt whatever that the institution at Bedminster, whatever its character, was under the proprietorship of the defendant, and was conducted under his direction and for his benefit. Some letters followed signed by Sister Hope, as matron. The prospectus was a very remarkable document, and it was headed "Bedminster Cottage Hospital and Training Home." There was a white cross on a black ground, and the motto "Ye did it unto Me." It was stated that "the institution was founded in September, 1897, in connection with the Nursery Aid Society, for the reduction of infantile misery and mortality. It was found that well-instructed midwives and nurses who would work among the people free, or at a nominal contribution, were necessary to carry that health teaching which is one of the special objects of the society's nurses into the actual homes of the people. Three months' training among the large number of out-patients (thirty weekly), and at the bedside of home patients (twenty-five to thirty weekly), fits the probationers for district nursing, and for entrance into hospitals or workhouses as probationers or assistant nurses, night nurses, &c. Two lectures are given daily, and practical instruction is given at the bedside in the hospital (five beds and two cots) or in the cottages of the people." The fee for general nursing and midwifery was £10 10s. for three months indoor. The staff was given as W. Ledingham Christie, M.D., F.R.C.S. Eng., resident surgeon and lecturer; Sister Hope, matron; the Misses Thompson and Willing, resident nurses. If the defendant had correctly stated the facts he would have described himself as the staff of the institution and as proprietor of a home which he thought the jury would have no doubt was opened for his own profit and advantage, and not for the profit and advantage of the poor people around. The correspondence satisfied the plaintiff, who sent £10 10s. on February 23rd, and Sister Hope sent a receipt. The plaintiff arrived in Bristol on March 1st and went to Bedminster, where she expected to find a cottage hospital. Instead of that she found Dr. Christie and Sister Hope occupying a portion of a house known as Church House, of which place Dr. Christie was a weekly tenant, paying 15s. a week. Instead of finding sanitary arrangements and appliances suitable for a hospital the plaintiff found there was no kitchen, the only accommodation for cooking being a stove in a cupboard near Dr. Christie's bed-room. The surgery was on the ground floor, and upstairs there was only one room, which was called a ward, and that contained two beds, one cot, and one cradle. There were no in-patients from January 19th until March 4th according to Dr. Christie's statement. There was a reference to outdoor patients in the prospectus, and the plaintiff would describe those she saw. Plaintiff's contention was that there was no cottage hospital in fact, and he should ask the jury to find that there was no cottage hospital in the sense which would be understood by people reading the advertisement and prospectus. The plaintiff was dissatisfied and left the institution, and she now claimed for the return of her money and compensation for loss and inconvenience.

Mr. Wansbrough, addressing the jury, said it was all very well for Mr. Inskip to depict what appeared to be a poor state of affairs at Church House, Bedminster, but that was not the question they had to decide. The charge made against the defendant was a charge of obtaining money under false pretences, and he contended they would have to look at the evidence in the same way as if they were trying a criminal case in the other Court on a charge of obtaining money by false pretences. It was a charge against a professional man, and a charge based upon a statement contained in the evidence given by the plaintiff and her one solitary witness. It was not a question of whether the house was comfortable, whether the rooms were large, or whether the food was good, but a question of whether there was fraudulent misrepresentation, a question as to whether the plaintiff could not have been trained if she had remained. The best answer to that would be found in the evidence of a number of ladies who had learned their profession with Dr. Christie, and who were now in situations and whom he would call. He thought he would be able to show that Dr. Christie conducted a *bone-fide* hospital and nursing home, where not only could the plaintiff have been taught her profession, but where a large number of nurses had been trained.

The Judge, in summing up, said the case was a most important one, though a small amount was involved. It was most important that it should be practically impossible for a bogus institution to exist which should divert into the pockets of those who ran it the earnings of people who could ill part with their earnings. That was one important view which the case presented from the view of Mr. Inskip. On the other hand it was an exceedingly important thing that a professional man should not have fixed upon him the stigma of fraud, nor should he have, unless they were clearly of opinion that he had committed a breach of contract, the stigma, so far as it went, of having broken the contract he made with a person in a comparatively helpless position like the plaintiff. The questions he put to the jury were: Did the defendant or did Sister Hope, when the letters and prospectus and advertisement were brought to the knowledge of the plaintiff, honestly believe that they were able to give to the plaintiff the training described in the letters, prospectus, and advertisement? Was the defendant ready and willing to give to the plaintiff substantially the training described in those documents? His Honour alluded to the dispute which Dr. Christie had in connection with the Children's Hospital, and asked the jury to dismiss entirely from their minds what they read of the inquiry into the matter.

The jury retired to consider their verdict, but were only away five minutes, and returned with answers in the affirmative to both the questions the Judge put to them.

His Honour: That is a verdict for the defendant.

### Minor Appointments.

Chester Ladies' Charity Institute.—Miss M. Hazlem Davies was appointed Matron on the 29th ult., and will enter upon her duties this month. She was trained at the Royal Infirmary, Liverpool, and has since held various appointments at home and abroad.

Millerton Fever Hospital.—Mrs. Ridehalgh has been appointed to the Matronship of the Millerton Fever Hospital, Ayton, Berwick-on-Tweed. She was trained at the Edinburgh Royal Infirmary, and has held appointments at the Hull Infirmary, South-Eastern Fever Hospital, and for the last eight years has acted as night superintendent of the Poplar and Stepney Sick Asylum, Bromley-by-Bow.

Hackney Union Schools.—Miss Lizzie Hutchinson was appointed Charge Nurse, May 24th. She was trained at Salford Union, and her previous appointments have been superintendent nurse at Holborn Union Schools, Mitcham, Surrey, and charge nurse at the Sheffield Union.

Infirmary of Hackney Union.—Miss Annie Jane Dashwood was appointed Charge Nurse, May 10th. She was trained at Middlesex Hospital. Her previous appointment was that of private nurse in connection with the London Association of Nurses, New Bond Street.

Wakefield Union Infirmary.—On May 17th Miss Lois Lightowler was appointed Sister. She was trained at Fir Vale Infirmary, Sheffield, and was afterwards night superintendent, Union Infirmary, Toxteth Park, and superintendent nurse, Gravesend Union, Infirmary.

Burnley Union Infirmary.—Miss Annie L. Charteris has been appointed Charge-Nurse. She was trained at Dumfries and Galloway Royal Infirmary, Dumfries.

### Where to Go.

Hotel Great Central.—Tuesday and Wednesday, June 6th and 7th, bazaar in aid of St. Mary's Hospital.

Victoria Hospital for Children, Chelsea.—Thursday and Friday, June 8th and 9th, bazaar in the garden.

Portman Rooms.—June 12th, at 3 p.m., Chevalier B. Palmieri's Matinee Musicale.

### Novelties for Nurses.

### THE "CELLULAR" GARMENTS.

Many of our readers, we believe, are wearers of the excellent corsets and underwear made by the Cellular Clothing Company, Fore Street, E.C. The garments manufactured by this company being so well known for durability and comfort as well as stylishness in appearance, other manufacturers have introduced numerous imitations of the original goods, many

THE CELLULAR CLOTHING Co. LTD. AERTEX LONDON

of these imitations being sold as "Cellular," and are of inferior quality. Under these circumstances any of our readers or their friends wishing to obtain the genuine and original "Cellular" should take care that the garments they buy bear the company's registered trade mark as here shown. Any garments sold without this registered trade mark are not the original and genuine article. The vital thing in the trade mark is the word Aertex in the centre.

### For Reading to the Sick.

Holy, Holy, Holy; Lord God Almighty! which was, and is, and is to come.—*Rev. iv.* 8.

Holy, Holy, Holy! Lord God Almighty!
Early in the morning our song shall rise to Thee
Holy, Holy, Holy, merciful and mighty,
God in Three Persons, Blessed Trinity.
Holy, Holy, Holy! though the darkness hide Thee,
Though the eye of sinful man Thy glory may not see;
Only Thou art Holy, there is none beside Thee,
Perfect in power, in love, in purity.

—*Bishop Heber.*

**Reading.**

From a belief in His own Person, Jesus Christ led the Apostles to a belief in the doctrine of the Holy Trinity. This belief grew out of the experience of the Apostles. Trained as Jews to believe in the unity of God, without losing their sense of the divine writers, they came, through the realisation of Christ as a divine Person, to recognise a plurality in the Godhead, and this belief was further enlarged as they learned about, and received the Holy Spirit. It is important to notice that the Apostles' belief in the doctrine of the Holy Trinity was a matter of experience, the outcome of their intercourse with Jesus Christ, and when they received the Holy Spirit on the Day of Pentecost their faith in the Father, the Son, and the Holy Ghost, as included in the being of God, received its full and satisfactory confirmation. . . . . While clearly proclaiming the unity of God, Jesus Christ revealed Himself as God's equal, and spoke of the Spirit, whom he was about to send from the Father, as a divine Person. . . . He commanded His Apostles, and their successors, to baptise all nations "into the name of the Father, and the Son, and the Holy Ghost." In this command He summed up the doctrine of the unity of God in a trinity of persons. Thus, Christ's language about Himself, the Father, and the Spirit is the basis of the Christian belief that there is one God in Three Persons.—*Staley.*

Father of Heaven, whose love profound
A ransom for our Souls hath found,
Before Thy Throne we sinners bend—
To us Thy pardoning love extend.

Almighty Son, Incarnate Word,
Our Prophet, Priest, Redeemer, Lord,
Before Thy Throne we sinners bend—
To us Thy pardoning grace extend.

Eternal Spirit, by whose breath
Our souls are raised from sin, and death,
Before Thy Throne we sinners bend—
To us Thy quickening power extend.

Thrice Holy! Father, Spirit, Son;
Mysterious Godhead, Three in One,
Before Thy Throne we sinners bend—
Grace, pardon, life to us extend.

### Presentations.

On the occasion of Sister Ridehalgh leaving the Poplar and Stepney Sick Asylum to take up work at Berwick-on-Tweed, she was presented by the medical staff with a handsome case of silver-mounted brushes, bearing a suitable inscription, and from the matrons and members of the nursing staff a silver-mounted purse containing gold, and a small testimonial neatly framed, as a tribute of esteem with which she is held. Mrs. Ridehalgh leaves the institution taking the good wishes of all her fellow-workers to her new sphere of work.

### A Book and its Story.

A NEW NOVEL BY A NEW WRITER.

THE readers who know Miss Tench's charming story, "Where the Surf Breaks," will not be surprised to find her latest novel* full of the same simple force which distinguished it. There is also pathos, humour, keen observation in the delineation of character, and a delicacy of style about her writing which is infinitely refreshing. We have but one fault to find with the construction of the present story, written with much vivacity and an underlying depth of tone which reveals the artist, and that is, the necessity of making two men propose to one sister when they were in love with the other! But if heroes propose rashly their authors can also dispose of them satisfactorily, so, in spite of these rather irritating little "contrarinesses," the story ends well.

And where lies "the land of the Great Never Never," from which the present book takes its title? Surely in some intangible mental region, known to most of us as one of unrealised aims and buried hopes! But no, it is a region real and tangible enough, in spite of its poetic name, bestowed by the natives of Central Australia, of which it forms a large and desolate tract. A poet, presumably an Australian, thus writes of this weird district:—

"Out on the wastes of the Great Never Never,
That's where the dead men lie!
That's where the earth's loved sons are keeping
Endless tryst; not the west wind sweeping
Feverish pinions can wake their sleeping
Out where the dead men lie!"

Into the desolate "Never Never country" sets forth the hero, Victor Carrington, in company with a party of scientific explorers. He is heir to a landed estate at home, and from sheer love of adventure has joined the expedition, having leisure and money at his command to further its interests. "To study the mental and physical aspects of the aborigines, their language and folk-lore, and later on to go farther afield to New Guinea and Timor, already visited by Wallace, was the intention of the explorers." To the ordinary Philistine the expedition seemed an absurd waste of time and money, and the opinion was one not unshared by more "enlightened" friends of Carrington's, who regretted that he should leave the duties and responsibilities attached to his position for the uncertain and perilous results which would probably attend the expedition.

Carrington's physique was one eminently adapted to bear the hardships of penetrating into an unknown country for "there was victory, or the promise of it, in the spare well-knit, upstanding figure, every line of the keen intelligent face, with its firm, rather thin-lipped mouth and clear light-grey eyes, every touch of the strong brown hand, every tone of the somewhat too incisive voice." A keen man and strong alike in character as in physique, which had earned for him the title of Achilles, given to him by his men friends, half in envy and half in derision of those very qualities which were named diversely pluck, cheek, "go-a-headedness, cock-sureness," according to the speaker's personal view of Carrington himself. It is during the voyage out that Molly Despard, travelling with an invalid uncle, meets "the Prince," who was to be the cause of so much pleasure and so much pain to her. "Achilles," of course, is "the Prince." Whatever motive besides that already named had been the cause of his joining the expedition, most certainly a woman was in no way concerned in it. For "as a matter of fact women as a rule bored him beyond measure." But in meeting with Molly, "so pure, so unselfish, so frank, so straightforward, everything in fact that was sweetest and most to be desired in woman, Achilles found himself touched in his vulnerable part in a manner which surprised him greatly." In her presence he was another man, courteous, gentle, deferential. The "cock-sure" manner so irritatingly displayed in the company of men forsook him entirely. Alas, it is ever thus! Molly having been a victim all her life to vacillation and want of vertebræ on the part of her own menkind, was attracted at once by the incisive, direct bearing which marked Victor Carrington's movements, and at once, dear little, faithful, soul that she was, adopted him as her hero.

"A Prince from the Great Never Never, with light touch of lips and of hand,
Had come and enslaved her for ever, a Potentate bearded and tanned."

After six weeks' daily companionship the moment of parting came at last, and "to her it seemed impossible that Victor Carrington was really in a few hours to go out of her life for ever; that the days would lengthen into weeks, the weeks into months, the months into years, and that his place would be empty, that he would not turn to her again with a genial smile, with kindly sympathy, with ready jest." They stood together on their last evening in the verandah of an hotel on Mount Victoria. The moon lit up the scene, and as they stood side by side a voice singing within the drawing-room thrilled out the words of an old song as new to-day in their simple, heart-breaking pathos as when they were written years ago—

"I cannot leave thee, tho' I said
Good-bye, sweetheart, good-bye."

But he did leave her, although he loved her deeply, with all the love his strong nature was capable of. Yet he never spoke, and, though looks are responsible for much, poor little Molly had to return and take up the unusually heavy burden of her daily life at the old home in Ireland, where a hopeless mother a still more hopeless father, and a frivolous sister, did their best to make her life unbearable. But with the memory of her brief love dream, and previous to that, bright days spent in India, still fresh upon her, Molly decides, if no one will make her happy, she, at any rate must set about brightening the lives of others. By her energy and self-suppression she soon begins to make the desert blossom as a rose; starts a flower farm, pays for her brothers' education, sets everyone up with a bicycle, and becomes herself a centre of light and sweetness, not only to her own circle, but to all the humble neighbours—her father's tenants, by whom she is adored. She brings together severed lovers, among other good deeds, and acts as "guardian angel" to the district. Then, very opportunely, when hope seems almost lost, her "Prince from the Great Never Never" returns, and after behaving in a foolish and "contrairy" manner, aggravating to a degree, but not unusual with heroes of the "Achilles" type, he marries dear little Molly, and everything ends comfortably. For all the fuller details of the romance our readers must go to the pages of this fascinating book itself.

* "A Prince from the Great Never Never." By Mary F. Tench. (London: Hurst and Blackett. 6s.)

### To Nurses.

IN order to increase and vary the interest in the *Mirror*, we invite contributions from any of our readers in the form of either an article, a paragraph, or information, and will pay a minimum of 5s. for each contribuion. All payments are made at the beginning of each quarter, *i.e.*, January 1st, April 1st, July 1st, and October 1st.

### Everybody's Opinion.

[Correspondence on all subjects is invited, but we cannot in any way be responsible for the opinions expressed by our correspondents. No communication can be entertained if the name and address of the correspondent is not given, as a guarantee of good faith but not necessarily for publication, or unless one side of the paper only is written on.]

### "DONT."

"A VERY OLD PRO." writes: One can hardly help smiling at the number of "Don'ts" that appeared in your paper the other day. I can hardly grasp the idea of a pro., however new she might be, mistaking an assistant matron for a fellow pro. Also the writer might explain the difference between a poultice-knife and a spatula. I was under the impression that they were one and the same thing, only differing in size. What does she mean by advising a pro. never to kneel down when on duty? In fact, others as well as myself fail to grasp the grit of a number of her "Dont's." Perhaps some of those who have had to undergo the "new pro." process will kindly give a sequel as to what a "new pro." may do. The writer of "Don't" must have gone through such an ordeal herself, as she so strongly recommends other probationers to be cautious.

### PRIVATE NURSES.

"JESMOND" writes from Newcastle-on-Tyne: I should just like to say a few words in defence of the much-maligned private nursing institute. I think, if nurses were careful to join only the long-established and thoroughly well-managed nursing institutes (and there are always vacancies on the staffs of these owing to a variety of causes), they would find in most cases that their lot was quite as happy, and certainly more secure, than that of their sisters who work on the co-operative system or on their own account. I know there are nursing homes, such as "Nurse B." mentions, where the nurses are merely part of the machinery, and where little or no interest is taken in them as individuals; but are not these the very institutions where the standard of efficiency required in those who join them is low? Do not these institutions accept nurses who have no three years' certificate—in many cases, indeed, nurses who have only had one year's hospital training—and is it to be wondered at that these places prove very unsatisfactory, both as regards the happiness and the financial position of those who join them? On the other hand, I think most nurses who belong to a nursing institution where a high standard of efficiency in the nursing staff is maintained, and where only thoroughly trained nurses are accepted, will bear me out in saying that every comfort is provided for them and every consideration shown them in case of ill health, &c. Not only is this the case in the nursing home to which I have the good fortune to belong, but I know that the matron takes a personal interest in the health and well-being of each individual member of the nursing staff, although it comprises nearly a hundred nurses. Surely Nurse B.'s experience of nursing institutions must be exceptionally unlucky if the nurses belonging to them have to go into lodgings during convalescence. My experience is quite the reverse, and I also know many private nurses both in Scotland and in the south of England whose experience of private nursing institutions is quite as happy as my own. May I suggest the advisability of all private nurses returning to hospital work periodically (say one year in every three) in order to keep abreast with all the latest improvements in nursing, treatment, &c. The knowledge one has already gained in hospital soon begins to get rusty unless being continually acted on and added to by actual experience, which it is obviously impossible to obtain while engaged as a private nurse.

"E. T. R." writes: As you have so kindly opened your columns to the contributions of nurses, I hope that the widespread influence of your valuable supplement may be the means of airing some of the profession's many grievances, and by helping to make nurses more co-operative—a spirit they seem greatly to lack—it may end, as the American says, in "a better mixin' all round." Then, perhaps, the Poor Law superintendent nurses will get their heart's desire and the private nurses justice. At present the latter are suffering from distinct libel, and being one myself I am in a position to judge. At the same time I am happy to be able to say that I belong to an old-established private institute in London, where our superintendent and his wife combine to make their nurses both happy and comfortable; where rules are few when "in"—three in number, I believe, and those unprinted—being left to our honour to keep, and therefore seldom broken; where a fixed salary is paid, indoor uniform provided, and a liberal sum weekly by way of commission is paid when at a case. Surely the heart of a private nurse could hardly desire more? We don't all spend our money on hansoms, Holborn Restaurant suppers, and theatre-going, and some of us even indulge in a P.O. bank book, help a little at home, and make one coat and skirt per annum in the way of private dress suffice. Certainly there are those among us whose one idea seems to be "a short life and a merry one," judging by their constant round of gaiety when "in," and yet I have seen these same nurses when on duty as alert and energetic as if pleasure were unknown to their busy minds. Personally nothing pleases me more when I am at liberty than to go and see a good play—not always half-crown seats either, and if my heart craved for a Holborn Restaurant luncheon I fear I should not think myself any the worse for such an indulgence, though I hope the "Mirror" will not be aghast at such reflections, but will agree with me that all work and no play makes Jill a very dull girl, and that an occasional treat does not mean ruination nor a destitute old age. It seems to me stupid to think that because one is a nurse life should be a long penance, as though one had taken the veil. We are not angels, and never shall be, and we are women, and, therefore, human. Let us take our pleasures while we may, like other people, provided we do our duty when it lies before us.

"A HAPPY AND CONTENTED NURSE" writes: With regard to those who grumble at private nursing institutions, I wish all who are discontented with their profession would give it up, and so leave the field to others ready perhaps to go into the work with heart and soul as did our beloved founder, Miss Florence Nightingale, who did so much to raise the standard of sick nursing. Nurse B. says that she is sure that the strongest objection to private institutions is that "nurses lose their individuality and simply become a part of the machinery." That may be her opinion, but I consider that a nurse connected with an institution has wider scope for cultivating her individuality than when in hospital or alone on her own account. Private nurses rub up against each other between cases, and can exchange views on different subjects. Nurse B. also complains that while in the "home" a nurse cannot go for a walk without permission. In most nursing "institutions" or "homes" that I know nurses are allowed to go out from ten till twelve in the morning, again from two to four; also, by permission from the matron, during the evening. A nurse working for herself or on the co-op, could not absent herself from her lodgings a longer time than two hours, or I am afraid doctor or patient would stand a poor chance of finding her at all. Surely, it is only due to a matron, at least, to let her know that a nurse will be absent. I have tried both working for myself and for a "nursing" home, and I am far happier in a "home" than in lodgings. I have been nursing ten years, and during that time I had a serious illness, and am happy to say I received every care and attention in my nursing institution, beside the matron paying my expenses at a convalescent home for me for three weeks. Life is very much what we make it, and if nurses are always ready to complain, instead of cultivating a spirit of thankfulness, it is difficult to sympathise with them. I have been nearly three years in my present post, viz., a "private nursing home," and can truly say there is not a happier home in England than ours. My fellow-nurses join with me in saying that if nurses only knew what the world and many members of the nursing profession are saying about the petty, mean, and discontented letters that have appeared in such a valuable paper as THE MIRROR they would be ashamed to think of the dishonour cast by them on one of the most noble callings for women.

### Travel Notes.

By Our Travelling Correspondent.

XXV.—FLORENCE—*(continued)*.

There are attractions for all tastes in the City of Lilies, and it is a singular fact that it exercises a powerful influence over nearly everyone, however, phlemgatic and matter-of-fact they may be. There are few who have once visited the entrancing city who do not long to return. Sometimes I think it is the life of the past so intimately mingled with our everyday concerns which is so fascinating; but, then, why do we not feel the same sensation in Rome? I must leave some more gifted person to explain the problem. When I first went there I was full to the brim of adoration of Venice, and felt but coldly towards Florence, but subsequent visits have changed all that.

### Architectural and Art Treasures.

I must not linger to speak much of these, the subject is so vast a one, and any good guide-book will give you all the information that you need; but I should like to warn you to be wary not to attempt to do too much. Sight-seeing is a killing process, and must only be taken in small doses. Now in the Uffizzi and Pitti galleries, for instance, those charming Italians are guilty of the brutality of allowing no chairs, except a few scattered far and wide in the corridors of the Pitti. Take a light camp stool with you, and thus be armed against nervous exhaustion; there are acres of pictures in these galleries, and the singular covered passage over the Ponte Vecchio, which connects the two, must be about a quarter of a mile long from the time it takes to traverse. The bridge itself is one of the curiosities of Europe; the jewellers' shops which line both sides remain as they were when the bridge was built, and in one of them on the left side, crossing to Oltrarno, Benvenuto Cellini lived and worked, and must have often swaggered up and down the Ponte Vecchio, handsome, proud, arrogant, and somewhat troublesome. The illustration I give you is from the Oltrarno end of the bridge, showing the tower of the Knights of Malta and the entrance to the Via di Bardi, where Romola lived. The churches are numerous and superb in Florence; visit them and the picture galleries in bad weather, and take the brilliant, sunny days for the many delightful excursions and drives in the neighbourhood.

### The Certosa di Val d'Ema.

You can visit this by steam tram, and it is a short excursion that will occupy only a morning. The charming garden, with the historic well, and the beautiful cloisters with the Lucca della Robbias, have suffered sadly from the earthquake of 1895. Fortunately it was very partial, only one side suffering. There are still a few white-robed monks remaining on sufferance. It has a melancholy effect on one to see so vast a building practically uninhabited. You buy delicious orris root powder here for perfuming clothes.

### Vallombrosa.

One can now go and return to Vallombrosa in one day, but it is a fatiguing affair and not to be recommended. If you do not wish to remain long, it is a good plan to spend from Saturday to Monday there; the woods are most delightful, and a few minutes in any direction will unfold to you fresh beauties. The convent is now used as a school of forestry, and it is strange to see the vast buildings once inhabited by the quiet monks now swarming with the life and spirits of the students. The Hotel Croce di Savoia, with its annexe, St. Paradisir o, gives good accommodation.

### Fiesole.

Fiesole lies some three and a half miles outside Florence on a lofty hill. The easiest way is to go by electric tram or to drive; fare 8 francs. There is a little restaurant, where one can get some indifferent coffee. The chief attractions are the ancient cathedral, very grim and grey, with a spacious crypt, which somewhat resembles that of San Zeno at Verona; the ancient theatre, with several rows of stone seats; and a portion of an Etruscan wall. These latter are behind the cathedral, and doubtless no properly-constituted mind will omit to visit them; for myself, I detest Roman and Etruscan remains, and had a full intention to shirk my responsibilities in this respect, but an energetic friend forcibly prevented this display of weakness, and not a stone or a seat was I permitted to gaze at perfunctorily. It was extremely cold I remember, and my thoughts turned affectionately to

At the Foot of the Ponte Vecchio.

the stuffy interior of the tram. The pleasures of Fiesole lie in quiet saunters on a fine mild day, far from energetic and conscientious friends, when one can be as lazy as one likes and enjoy the magnificent views all round, more especially that from the Franciscan monastery far above the Cathedral.

### The Cascine and San Miniato.

These two localities are at the opposite ends of the city. The rank and fashion of Florence disports itself in the Cascine gardens, especially on Sundays when the military band plays. The gardens are stately and very pleasant, and well suited to those unable to walk much. When you visit San Miniato let it be when there is a good sunset if possible, that is the ideal moment when the City of Lilies lies below bathed in crimson and gold. At first I felt much affronted with a replica of Michael Angelo's "David," which is placed on the platform below the church, but I got accustomed to it, and felt that, like everything else in Florence, it was the right thing in the right place. The Boboli Gardens are interesting as showing what landscape gardening was in the time of Cosimo I. Here, too, at sunset, is an entrancing view of the Duomo, Giotto's tower, &c., a view so popular that the moment one sees it one is conscious of encountering an old friend.

SAVONAROLA.

You will doubtless very early seek out the places made for ever famous by the footsteps of Girolama Savonarola, the convent of San Marco, where he lived and suffered; the adjoining church, where his burning words went home to the hearts of thousands who thronged to listen to the prophet till the space proved too limited and he moved into the Duomo and preached against the sin, the luxury and the guilt of all classes; the Palazzo Vecchio where he was imprisoned; and the Palazzo where he suffered martyrdom. All these places speak of him, but to me, the spot where I most realise his dauntless presence is in the Villa Careggi outside Florence, where the great Lorenzo died, and whither he summoned Savonarola to absolve him. This he refused to do unless the Magnifico restored the liberties of Florence. It must have been a strange and impressive scene, Lorenzo died unabsolved, and those two powerful and haughty men met for the last time.

PISA.

Pisa may be visited without much fatigue in one day from Florence, and it is not a very interesting city in which to stay unless you are an artist. The principal buildings, as most people know, are all close together, as if conveniently gathered together for the inspection of visitors. The cathedral is grand, and if one has not already seen Siena it proves very attractive. The Baptistery is rich and gorgeous, but does not appeal to me; there seems a sort of nakedness about it, all alone and unsupported by other buildings. I prefer that of Florence. The leaning tower is a marvel, and if you should chance to see it when an east wind is blowing (no uncommon occurrence in Pisa), the atmospheric effect on the mountains behind, leaving them vividly blue, and throwing up the tower as if it were constructed of snow, is very striking. My affections turn most fondly to the Campo Santo on account of the delightful frescoes, especially the "Hermits." I have spent hours studying the artless representations of those holy men, their difficulties, their temptations, and their domestic arrangements.

### TRAVEL NOTES AND QUERIES.

ST. MALO OR THE ARDENNES (Firenze).—If you go to the Bay of St. Malo, Madame Pallot, Maison Massias, St. Servan, or Miss Humphreys, Rue Le Pomellic, will take you for from six to seven francs a day—seven francs is 5s. 10d. At Paremé the Hotel Continental, the Hotel des Bains, and Hotel du Centre will ask the same terms. Write beforehand. You start from Waterloo and take the boat at Southampton. No reduction on a tourist ticket. (2) For the Ardennes you start from Liverpool Street, and go via Harwich and Antwerp. Get your ticket from Cook, Gaze, or Dr. Lunn, and you will have the cheapest available at the season. At Dinant the Hotel Tête d'Or, 7½ francs pension, is very pleasant; the Hotel des Familles is cheaper, six to seven francs, and very comfortable. It is impossible to say what the expenses of your excursions will be because I don't know where you wish to go; but they are all cheap, from one to three francs covering the outlay, except that to Mont St. Michel, which, including a night on the mount, will cost from 15 to 20 francs. Any month is suitable, but things are dearer and hotels most crowded in August and September.

BOARDING HOUSE IN BRUSSELS (Winifred).—A reasonable pension is that of S. Bernard, 50, Rue Belliard, seven francs a day; and Madame Schümann, 14, Rue d'Orleans, seven to eight francs. A cheap German hotel is the Hotel de Bavière, pension six francs, and another, Hotel du Rhin, 14, Rue de Brabant, six francs. Write beforehand and make terms. Ask for rooms on third floor. At the Hague there is a very cheap pension called S'gravenhaag'sche Pension Maatschappy, 26, Yavastraat. Write and ask terms. They range from 2½ florins, which is 5s., if you remain an entire week or longer.

BERLIN FOR A WEEK OR TEN DAYS (Frank).—The cheapest route is viâ Harwich and the Hook of Holland—return ticket for 30 days, second-class, £4 1s. Only one night on the water. You need not sleep on the journey. You land at five o'clock in the morning, go on at 5.45, and reach Berlin at 10.45 that night. Take food with you; it is very dear on the rail, and unless you speak German you might find difficulties. I do not think you will find officials speaking English, but write the address in Berlin on a piece of paper plainly, and have it at hand, and your ticket will show your destination. Everyone is very kind to a foreigner; there is nothing to fear. As to tips, they are much higher than in England; where we should give twopence in England you must give sixpence abroad. Get your ticket at Cook's or Gaze's, and have your money changed there. German coins are what you want, and a very little Dutch. Yes, you can take your portmanteau, if small, in with you.

*(For Travel Advertisements see Page* xvi.*)*

### Notes and Queries.

**The contents of the Editor's Letter-box have now reached such unwieldy proportions that it has become necessary to establish a hard and fast rule regarding Answers to Correspondents. In future, all questions requiring replies will continue to be answered in this column without any fee. If an answer is required by letter, a fee of half-a-crown must be enclosed with the note containing the enquiry. We are always pleased to help our numerous correspondents to the fullest extent, and we can trust them to sympathise in the overwhelming amount of writing which makes the new rules a necessity.**

**Every communication must be accompanied by the writer's name and address, otherwise it will receive no attention.**

(88) "G. B." asks: Being a trained nurse and holding L.O.S. diploma I am engaged by an institution as district midwife. Should I deliver a breech in a primipara and any complication occurs, who would be held responsible? My L.O.S. diploma forbids me taking such a case on my own responsibility, except in a multipara, without medical assistance.

If you do what your certificate forbids you to do there can be no doubt that you would be held responsible in case of accident.

*Army Nursing Abroad.*

(89) Will you kindly tell me how and where I can get information regarding soldiers' nurses? I wish to be a Government nurse to go abroad (I prefer India). I would like to go out soon. I shall be very grateful for any information you can give me. I love my work and have plenty of courage for it.—*Bon Voyage.*

You will find full information on all the nursing services under Government Departments in "The Nursing Profession: How and Where to Train," price 2s., from the Scientific Press, London, W.

*Medical Fees in Cottage Hospitals.*

(90) In answer to the letter in this week's HOSPITAL on this subject, I should like to state that the doctors in this neighbourhood send patients into our nursing institution, and receive their own fees from the patients. The institution was opened last July, and the arrangement seems to work well. We have four beds and one cot, and we have admitted 27 patients since the opening. It bears the name of "institution," but it does the work of a cottage hospital with a large district work attached. I have worked amongst cottage hospitals for years, but I must say that I never before heard of doctors receiving fees from patients.—*Nurse-Matron.*

*Probationer.*

(91) Can any reader inform me of a hospital where I could be trained as a nurse; my age 21, but height barely 5 ft?—*May.*

Why not consult our advertisement columns? You are not quite tall enough to be acceptable to some matrons, and are still rather young. You would also find "The Nursing Profession: How and Where to Train" (price 2s.), from the Scientific Press, most useful.

*Massage.*

(92) I shall be glad if you will kindly inform me where I could get the best training in massage, and cost of training. I am considering taking it up owing to my sight failing, and am anxious to secure the best certificate obtainable.—*H. W. M.*

We see that Mrs. Creighton Hale, the author of the "Art of Massage" (published by the Scientific Press), makes tutelage of the blind a speciality. Her address is 89, Mortimer Street, London, W. Write and ask her to quote inclusive terms.

*Bath Chair.*

(93) Is it customary for trained nurses to wheel the Bath chair of invalids? Ought they to be expected to do so?

This is certainly a question of private arrangement between patient and nurse. Wheeling Bath chairs cannot be said to come under the head of a nurse's duties.

*The L.O.S. Examination.*

(94) "S. E. H." wishes to obtain her L.O.S. Is it possible to do so at any Poor-law infirmary or any hospital without heavy fees?

It is possible to pass the L.O.S. examination from any hospital or workhouse infirmary from which a certificate can be obtained of having attended the necessary number of confinements according to the regulations of the Obstetrical Society.

*Tuberculosis.*

(95) "Nil Desperandum" asks for the authors and publishers of any books, English or foreign, on the "Nordrach" cure of tuberculosis. Also whether the cure is one for tuberculosis of the lungs only?

The latest information as to the various existing sanatoria can be obtained from a book recently published by Dr. Rufenacht Walters on "Sanatoria for Consumptives" (Swan Sonnenschein and Co.) We believe that Nordrach is a "closed" sanatorium—that is, one in which consumptive cases only are received.

*A Question of Authority.*

(96) Has the superintendent of a nursing institution (who is not a nurse) legal authority to forbid one of her nurses to visit certain people during her off duty time, and to instantly dismiss her without paying her a month's salary, or even that which is due for the previous three weeks on her disobeying that command?—*Nurse.*

It is impossible to judge such a case in the abstract. Consult a lawyer as to the best way in which to recover the salary due.

*The Children's Nurse.*

(97) In one of your numbers last year I saw the address of a home in London where girls are trained as children's nurses, and afterwards found situations. Will you kindly give it?—*Roderic.*

The address you ask for is the Norland Institute for Training Children's Nurses, 29, Holland Park Avenue, Notting Hill, W.

SUPERFLUOUS HAIRS.—We have frequently had inquiries regarding the removal of superfluous hairs. Perhaps some of our correspondents may be interested in the article which appears in THE HOSPITAL on page 163.

## Figures and Tables

**Figure f1:**
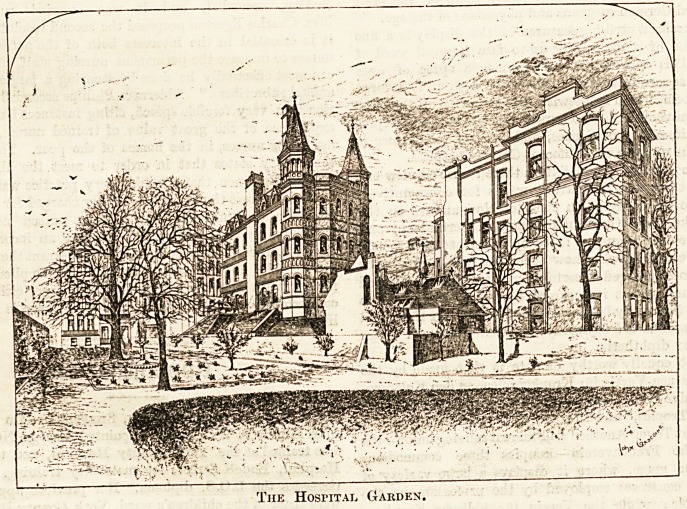


**Figure f2:**
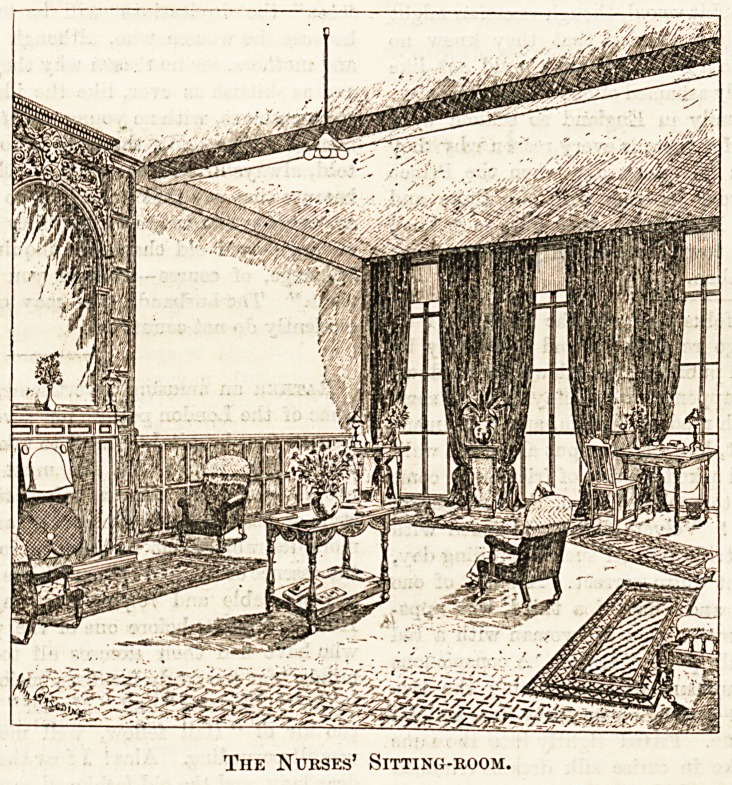


**Figure f3:**